# Hyper‐buoyancy flotation increases cervical disc height and reduces vertebral stiffness, with only partial reversal after acute 1 *g* axial loading

**DOI:** 10.1113/EP093601

**Published:** 2026-06-05

**Authors:** D. Marcos‐Lorenzo, P. Hernández Bernal, R. Fernández Corujo, A. Gil Martínez, J. Swanenburg, D. A. Green

**Affiliations:** ^1^ School of Medicine Autonomous University of Madrid Madrid Spain; ^2^ Department of Physiotherapy Centro Superior de Estudios Universitarios La Salle Madrid Spain; ^3^ CranioSpain Research Group Centro Superior de Estudios Universitarios La Salle Madrid Spain; ^4^ Unit of Physiotherapy Hospital Universitario La Paz‐Carlos III, Institute for Health Research, IdIPAZ Madrid Spain; ^5^ Faculty of Medicine Institute of Aerospace Medicine, University of Zurich Zurich Switzerland; ^6^ Integrative Spinal Research ISR, Department of Chiropractic Medicine Balgrist University Hospital Zurich Switzerland; ^7^ Centre of Human & Applied Physiological Sciences (CHAPS) King's College London London UK; ^8^ Institute for Risk and Disaster Reduction University College London London UK

**Keywords:** cervical spine, hyper‐buoyancy flotation, intervertebral disc, microgravity analogue, neck pain, vertebral stiffness

## Abstract

Exposure to microgravity is associated with stature increases, moderate‐to‐severe back/neck pain and elevated lumbar and cervical intervertebral disc (IVD) herniation risk post‐flight. Whilst lumbar pathophysiology has been investigated, little attention has been placed on the cervical spine. Thus, the aim of this study was to determine the effect of 4 h of hyper‐buoyancy flotation (HBF) and subsequent 15 min of upright 1 *g* exposure on stature, cervical IVD height, cervical muscle cross‐sectional area (CSA) and thickness, passive cervical vertebral stiffness, and their association with stature changes and/or neck pain. To investigate this, 12 healthy volunteers (five male; 30.2 ± 7.2 years, 66.1 ± 11.7 kg, 173.4 ± 8.5 cm) participated. Stature was assessed at Pre HBF, after 4 h of supine HBF (Post HBF), and following 15 min of upright 1 *g* exposure (Post 1G). Cervical IVD height (C3–T1), longus colli and semispinalis cervicis CSA and thickness, passive vertebral stiffness (C2–L5), and neck pain were assessed at Pre HBF, HBF0, HBF2, HBF4 and Post 1G. Results indicated that 4 h HBF significantly increased stature (+1.6 ± 0.5 cm), which was partially reduced after acute 1 *g* exposure (−0.4 ± 0.3 cm). Cervical IVD height increased at all levels following HBF, with partial reversal at Post 1G, particularly in the lower cervical region. Longus colli and semispinalis cervicis CSA and thickness were unchanged. Passive vertebral stiffness significantly decreased (across the entire column and most regions) following HBF and was only partially restored after 1 *g* exposure. Neck pain increased during HBF and was reduced after 1 *g* exposure. No associations were observed between neck pain and stature or IVD changes. However, neck pain at HBF4 was positively correlated with mid‐cervical vertebral stiffness (*R* = 0.587, *P* = 0.045). Overall, 4 h HBF induced mild neck pain, increased stature and cervical IVD height, all of which were partially reduced after 15 min of upright 1 *g* exposure. No changes in longus colli or semispinalis cervicis muscle thickness or CSA were observed. Passive cervical vertebral stiffness decreased following HBF and remained below baseline after 1 *g* exposure. A positive correlation between neck pain at HBF4 and mid‐cervical vertebral stiffness suggests modulation of vertebral column stabilisation may contribute to symptoms. The lack of association between neck pain and IVD expansion suggests that vertebral flattening may play a role and therefore warrants direct evaluation with HBF and following spaceflight.

## Abstract (highlights)

- How do 4 h of hyper‐buoyancy flotation and subsequent 15 min of upright 1 *g* exposure affect stature, cervical IVD height, cervical muscle morphology, passive vertebral stiffness and neck pain?
- Four hours of HBF increased stature and cervical IVD height, reduced passive vertebral stiffness and induced mild neck pain, and this was only partially reversed by 1 *g* exposure. Longus colli and semispinalis cervicis CSA and thickness remained unchanged. Neck pain correlated with mid‐cervical vertebral stiffness, suggesting altered vertebral stabilisation. These findings highlight potential mechanisms underlying microgravity‐induced neck pain and provide new insight for targeted countermeasures in astronauts and future HBF research.

## 1 INTRODUCTION

Spinal unloading in microgravity is associated with stature increases of up to 7 cm (Brown, 1977) and moderate‐to‐severe back pain in more than half of astronauts (Wing et al., 1991). Whilst the majority report pain in the lumbar region, a significant proportion of crew report cervical (neck) pain (Pool‐Goudzwaard et al., 2015). The specific pathophysiology underlying such spinal changes is unknown (Green & Scott, 2018) despite spaceflight being associated with intervertebral disc (IVD) swelling (Sayson & Hargens, 2008), spinal curvature flattening (Andreoni et al., 2000), trunk muscle atrophy (Sayson et al., 2013) and reduced para‐spinal muscle tone (McNamara, Greene, Moore et al., 2019). Despite extensive study, it is unknown how such changes contribute to an apparent increased risk of lumbar and cervical IVD herniation post‐flight (Johnston et al., 2010).

Increased stature has led to several astronauts having difficulty fitting into their extra‐vehicular activity (EVA) suits, and the bespoke Soyuz Kazbek seat pan (Nicogossian et al., 2016). However, despite the operational significance, few spinal studies have been performed inflight – in part due to the challenges of medical imaging (Green & Scott, 2018). However, magnetic resonance imaging (MRI) has been performed following long‐duration (≥6 months) International Space Station (ISS) spaceflight, although most research has focused on the lumbar region (Bailey et al., 2022; Chang et al., 2016; Sayson et al., 2013). Significant lumbar IVD pathology has been reported (Chang et al., 2016; Sayson et al., 2013), in addition to multifidus atrophy and impaired spinal segment kinematics (Bailey et al., 2022), consistent with vertebral dysfunction (Hides et al., 2021). Interestingly, multifidus atrophy differed between the upper and lower lumbar segments, whilst impaired spinal segment kinematics were associated with post‐flight lumbar disc herniation and pre‐existing spinal endplate irregularities (Bailey et al., 2022) suggestive of spinal load sharing dysfunction (Breen et al., 2023).

Inflight ultrasonic evaluation on the ISS (Marshburn et al., 2014) identified a range of lumbar and cervical IVD pathologies, including disc desiccation, osteophytes and endplate sclerosis (Garcia et al., 2018). Despite the observation of cervical pathology both inflight (Garcia et al., 2018) and during offloading (Belavy et al. 2016) and the fact that the cervical spine is reported to be 21.4‐fold more vulnerable to post‐flight IVD herniation compared to matched controls (Johnston et al., 2010), cervical pathology has received remarkably little attention.

However, whilst long‐duration 6° head‐down tilt bed rest (HDTBR) is reported to induce relatively minor lumbar spine modulation (Hutchinson et al. 1995), increased upper and mid‐thoracic spine IVD height and volume has been reported (Belavý et al., 2013). In fact, cervical muscle (splenius capitis, spinalis cervicis, longus capitis, longus colli, levator scapulae, sternocleidomastoid and the scalenes, but not semispinalis capitis) hypertrophy has been observed following 60‐day HDTBR (Belavý et al., 2013). This may be due to head weight re‐orientation and weight‐bearing as it contrasts with a tendency to induce either mild cervical muscle atrophy (LeBlanc et al., 2000) or maintenance post‐flight (McNamara, Greene, Tooze et al., 2019). In the same study significant increases in trapezius, semispinalis capitis, sternocleidomastoid and rhomboid minor muscle CSAs were also reported (McNamara, Greene, Tooze et al., 2019). A potential explanation for such findings is that whilst microgravity is associated with unloading, significant neck muscle activation may still be induced during activities such as exercise countermeasures (Green & Scott, 2018) – whose capabilities (and thus loading) have been upgraded over time (Scott et al., 2019).

For instance, the increased running speed during treadmill running was facilitated by provision of the T2 treadmill, followed in 2009 by implementation of the Advanced Resistive Exercise Device (ARED) that utilises pressure cylinders to provide resistance‐type exercises (Petersen et al., 2016). Whilst the T2 treadmill provides relatively sustained shoulder loading of approximately 0.7 g through the Glenn Harness during aerobic countermeasure sessions, ARED use presumably induces high impulse loading through the shoulders (Green & Scott, 2018). Therefore, spinal status during and after long‐duration spaceflight reflects not only unloading, but also intermittent axial loading (Green & Scott, 2018). Interestingly, Belavý and co‐workers reported that two participants presented mid–lower thoracic injury evident on MRI post‐HDTBR, having performed resistive vibration exercise countermeasures (Belavý et al., 2013). Thus, whilst post‐flight MRI may be informative, they may also reflect loading forces during re‐entry, landing and re‐ambulation in 1 *g* – particularly as the imaging is not usually performed until at least 24 h, but more frequently 48 h following landing (Bailey et al., 2022).

Change in spinal loading (and load sharing) may affect muscle geometry reflected in size (e.g., CSA) and/or tone/contractility (e.g., muscle thickness). In fact, changes in (relatively superficial) cervical muscle such as semispinalis cervicis and longus colli (Franchi et al., 2018; Maughan & Nimmo, 1984) have been suggested to be indicative, which is intriguing as the modulation of semispinalis cervicis and longus colli has been associated with chronic idiopathic neck pain on Earth (Looveren et al., 2021).

Whilst muscle atrophy takes time to manifest, significant lumbar curvature flattening such as that reported post‐flight (Andreoni et al., 2000) has also been reported in the upper lumbar and lower thoracic spine during transient microgravity induced by parabolic flight (Meinke et al., 2025). An association with attenuated multifidus and erector spinae activity is suggestive of rapid neuromuscular control adaptation and potential spinal re‐alignment (Meinke et al., 2025).

Interestingly, patients with chronic neck pain are commonly observed to express cervical IVD expansion and fat infiltration in multifidus, semispinalis (Schomacher et al. 2013) and longus colli (Grondin et al., 2022), suggestive of muscular dysfunction. Changes in load sharing and muscle recruitment may modulate active vertebral stiffness – defined as the vertebral column's deformation in response to imposition of a displacement force (Glaus et al., 2021). As vertebral stiffness on Earth is posture (gravity) dependent (Häusler et al., 2020), it is defined as ‘passive’ when supine/prone, and ‘active’ when upright, and thereby weight‐bearing (Stokes & Gardner‐Morse, 2003).

Recent parabolic flight studies have reported rapid increments in active lumbar (L3) vertebral stiffness during transient (∼20 s) microgravity (Swanenburg et al., 2020), and simulated (0.16 *g*) lunar and Martian (0.37 *g*) gravity (Swanenburg et al., 2023), with commensurate reductions during hypergravity (∼1.8 *g*) (Swanenburg et al., 2020). However, in another study, a more spatially nuanced response to axial loading was observed, with decreased vertebral stiffness in the upper cervical region, increments in the lower cervical region, and no change in the middle cervical region (Hofstetter et al., 2021). A complex pattern of vertebral modulation including a significant decrease in both active and passive thoracic vertebral stiffness, and a tendency for cervical stiffness to decrease is also observed following performance of trunk exercises when supine but exposed to 1 *g* at the centre of mass generated by short‐arm human centrifugation (SAHC) (Marcos‐Lorenzo et al., 2022). Yet, to our knowledge (passive) cervical vertebral stiffness has yet to be evaluated inflight, on SAHC or in a ground‐based microgravity analogue that unloads the cervical region.

Whilst HDTBR is the most commonly employed ground‐based microgravity analogue, as has been described above it is sub‐optimal as a model of spinal changes in microgravity (Hargens and Vico, 2016) with a lower prevalence of back pain, stature increments no greater than 8 h of normal sleep (Styf et al., 1997) and trunk musculature changes inconsistent with spaceflight‐related data (Hargens and Vico, 2016). In contrast, dry immersion (DI), based on the subject being ‘immersed’ in a water‐filled bath whilst lying on flexible waterproof material allowing them to remain dry but suspended, is reported to rapidly induce lower back pain (Rukavishnikov et al., 2017; Tomilovskaya et al., 2019) and modulation of vertebral muscle tone (Plehuna et al., 2022). However, stature increments are modest (Treffel et al., 2017), and DI is unsuitable for evaluation of the cervical region as the head is supported out of the water to negate motion sickness, which presumably induces significant cervical vertebral loading and resultant muscle activation.

A novel ground‐based analogue of spinal unloading was recently developed at King's College London to support evaluation of the SkinSuit programme prior to evaluation in spaceflight (Stabler et al., 2017), termed hyper‐buoyancy flotation (HBF) (Breen et al., 2023). HBF involves subjects lying on a waterbed which is partially filled (∼50%) with water supersaturated with sulphate of magnesium (Epsom salts) such that body segments, including the head, sink into the bed creating a ‘passive’ longitudinal posture (Marcos‐Lorenzo et al., 2024). Eight hours of HBF has been shown to induce significant increases in stature (2.1 ± 0.2 cm), and was associated with moderate lumbar and cervical, but not thoracic, back pain (Carvil, Russomano, Halson‐Brown et al., 2017). Four hours of HBF has also been demonstrated to induce substantial (albeit lower) stature increments (1.8 ± 0.2 cm) and mild back pain (Carvil, Russomano, Halson‐Brown et al., 2017). Furthermore, 4 h HBF has recently been reported to increase lumbar IVD height and moderate back pain, which were partially ameliorated by both 15 min upright sitting and 30 s of 50% BW axial loading (Marcos‐Lorenzo et al., 2024). However, whether acute HBF induces significant changes in cervical IVD height, passive cervical vertebral stiffness, cervical muscle CSA and thickness, how they may relate to stature increments and neck pain, and their reversibility with re‐exposure to gravity alone are unknown.

Thus, the aim of this study was to determine the effect of 4 h of HBF and subsequent 15 min of upright 1 *g* exposure on cervical IVD height, cervical muscle CSA and thickness, passive cervical vertebral stiffness, and their association with induced stature increments and/or neck pain.

## 2 METHODS

All procedures performed in studies involving human participants were in accordance with the ethical standards of the institutional and/or national research committee and with the 1964 *Declaration of Helsinki* and its later amendments or comparable ethical standards. This project was approved by the CSEU‐Lasalle Ethics Committee, code CSEULS‐PI‐032/2020, and registered on 25/03/2022 at ClinicalTrials.gov (NCT05296200). All participants provided written informed consent prior to their inclusion in the study. Written informed consent was obtained from all the participants for publication of the data and images in an open access journal.

Twelve (five male) healthy volunteers (30.2 ± 7.2 years, 66.1 ± 11.7 kg, 173.4 ± 8.5 cm) who conformed with current NASA astronaut anthropometric requirements (1.57–1.90 m, 50–95 kg) (NASA, 2025) participated in this study. Prior to inclusion in the study, all subjects completed a medical screening including exclusion of current back/neck pain, musculoskeletal disorders, cardiovascular disease, spine surgery, or actual pregnancy or suspected pregnancy. All subjects were recreationally active and reported engaging in sport (on average) at least twice per week.

**FIGURE 1:**
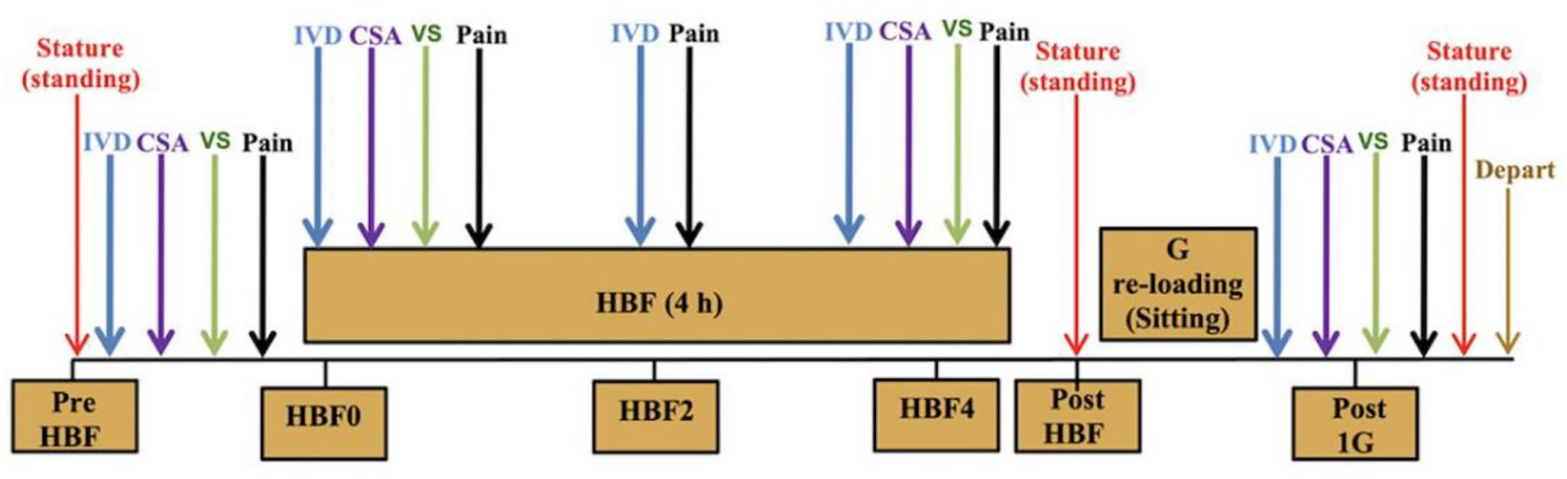
Schematic representation of the experimental protocol. Standing stature was assessed at Pre HBF, immediately after 4 h of hyper‐buoyancy flotation (Post HBF), and following 15 min of upright 1 *g* exposure (Post 1G). Cervical IVD height via ultrasound (IVD), longus colli and semispinalis cervicis cross‐sectional area (CSA) and thickness, passive vertebral stiffness (VS), and neck pain were recorded at Pre HBF, HBF0, HBF4, and Post 1G. Cervical IVD height and neck pain were additionally recorded at HBF2.

Participants attended the laboratory at IRF of CSEU La Salle in Madrid (Spain) on a single occasion. Standing stature was determined prior to (Pre HBF), immediately after 4 h of supine HBF (Post HBF), and following 15 min of upright 1 *g* exposure (Post 1G) (Figure 1). Cervical IVD height, muscle CSA and thickness, passive vertebral stiffness, and neck pain were recorded prior to (Pre HBF), and immediately upon lying on the HBF (HBF0) in addition to immediately prior to rising (HBF4) and following the 15 min of upright 1 *g* exposure (Post 1G). Throughout the paper, ‘1 *g* exposure’ refers to the 15 min period of upright sitting under normal gravitational loading, whereas ‘Post 1G’ refers to the post‐exposure assessment time point. Participants were instructed to remain supine and motionless, except when passively re‐orientated by the experimenters to and from the prone position to facilitate semispinalis cervicis ultrasonic and passive vertebral stiffness assessment (<2 min). Cervical IVD height and neck pain were recorded at HBF0, HBF2 and HBF4.

Subjects lay supine upon a HBF waterbed (Marcos‐Lorenzo et al., 2024) encased within a wooden frame partially (50%) filled with water supersaturated with magnesium sulphate (1.7 g cm^−^^3^) rendering participants buoyant, albeit sinking passively into the bed in proportion to their segmental mass. Water temperature was regulated by a conductive heater placed underneath the bed generating a thermoneutral temperature (34–36°C) and thereby maintaining passive thermal comfort.

**FIGURE 2:**
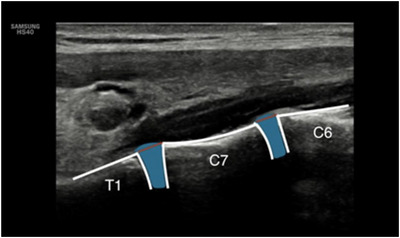
Longitudinal plane ultrasonic image of C6–C7 and C7–T1 cervical intervertebral disc (IVD) spaces with markers placed at the most superior anterior point of the inferior vertebrae and the most inferior anterior point of the superior vertebrae to facilitate anterior IVD height assessment.

Standing stature was determined with a commercially available stadiometer (Seca 217, Seca GmbH & Co. KG, Hamburg, Germany), whilst cervical IVD height from C3 to T1 (C3–C4, C4–C5, C5–C6, C6–C7, C7–T1) was assessed when supine via portable ultrasound (HS40; Samsung Medison Co., Ltd., Seoul, Republic of Korea) with a linear array (6–12 MHz) similar to that performed on the ISS (Marshburn et al., 2014). The probe was orientated para‐sagittal to the oesophagus starting at the manubrium and worked superiorly up the neck to visualize the upper cervical IVDs, with mild compressive force applied by the investigator to obtain optimal images. Markers were placed at the most superior anterior point of the inferior vertebrae and the most inferior anterior point of the superior vertebrae with the inter‐point distance taken as the anterior IVD height. At least two longitudinal plane images per level were acquired (at each time point) to ensure IVD height repeatability (Bland & Altman, 1986) (Figure 2).

**FIGURE 3:**
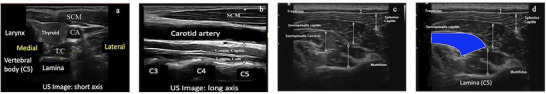
(a) Longus colli (LC) cross‐sectional area (CSA) and (b) muscle thickness; (c) semispinalis cervicis muscle thickness and (d) CSA in transverse view measured as the greatest distance from border‐to‐border excluding connective tissue at the level of C5.

Longus colli and semispinalis cervicis CSA and muscle thickness were also acquired with the linear array ultrasound probe. Longus colli CSA and muscle thickness were determined with the subject lying supine with both arms resting along the sides of the body, and the head in a neutral position. The ultrasonic probe was placed perpendicular to the vertical axis of the neck to generate an axial image (Figure 3a) (Javanshir et al. 2011) after identifying the thyroid cartilage and moving 2 cm inferiorly from which longitudinal images were obtained to assess relaxed muscle thickness (Figure 3b).

Semispinalis cervicis CSA and muscle thickness were determined with subjects lying in a prone position. The transverse process on the lateral aspect of C7 was identified in the transverse plane. The ultrasonic probe was then moved medially to visualise the lamina and turned longitudinally to identify the cervical facet joint of C5–C6. The articular facet between C5 and C6 was placed in the centre of the image and the probe was again turned transversely (Øverås et al., 2017) from which muscle thickness (Figure 3c) and CSA were acquired (Figure 3d).

IVD anterior height, muscle CSA and thickness were analysed offline via dedicated software with the ImageJ program (LOCI, University of Wisconsin, Madison, WI, USA).

Passive vertebral stiffness from C2 to L5 was assessed in the prone position with a handheld differential vertebral stiffness transducer (PulStar, Sense Technology Inc., USA) manually placed and held perpendicularly upon each vertebral spinous process. A preload >18 N triggered the measurement, which compresses the soft tissue between the transducer head and the target spinous process (Hofstetter et al., 2018; Leach et al., 2003). Thus, the triggered impulse propagation properties reflect vertebral stiffness (Girod et al., 1997) captured via proprietary software (PulStar, Sense Technology Inc., Halifax, PA, USA). Resultant vertebral stiffness (C2 to L5) possesses good test–retest reliability even with trained novice examiners (Hofstetter et al., 2018), with excellent reliability across the spine, including the cervical region (Hofstetter et al., 2021). Body pain ratings were acquired using the CHAPS body pain rating scale used in previous HBF studies (Marcos‐Lorenzo et al., 2024) to evaluate neck (cervical) pain.

### 2.1 Data analysis

All data were normally distributed (Shapiro–Wilk test); thus, the effect of load (HBF‐induced unloading and 1 *g* exposure) was assessed by ANOVA followed by a *post hoc* Student's *t*‐test (Bonferroni corrected). The effect of load on standing stature was assessed with a repeated measures ANOVA (Pre HBF, Post HBF and Post 1G). The effect of load on IVD height was assessed with a repeated measures ANOVA (Pre HBF, HBF4, Post 1G). Subsequently, the specific effect of HBF (HBF0, HBF2, HBF4) was assessed with a repeated measures ANOVA. The effect of load on longus colli and semispinalis cervicis muscle CSA and thickness was assessed with a repeated measures ANOVA (Pre HBF, HBF4 and Post 1G). Effect of load on passive vertebral stiffness was evaluated by a repeated measures ANOVA (Pre HBF, HBF0, HBF4, Post 1G). The effect of loading (Pre HBF, HBF0, HBF2, HBF4, Post 1G) on neck pain was evaluated by a repeated measures ANOVA. The associations between induced stature, neck pain, cervical IVD height and passive vertebral stiffness changes were evaluated by Pearson's correlation coefficient in response to 4 h HBF unloading and 1 *g* exposure.

All data are reported as means ± standard error of the mean (SEM), together with 95% confidence intervals (CI). All statistical tests were performed using IBM SPSS version 21 (IBM Corp., Armonk, NY, USA) with statistical significance set at *P* < 0.05.

## 3 RESULTS

All participants completed the 4 h HBF protocol and the subsequent 15 min of 1 *g* exposure without issue.

### 3.1 Standing stature

Standing stature increased significantly following HBF (+1.6 ± 0.5 cm) and was partially reduced after 1 *g* exposure (−0.4 ± 0.3 cm), although it remained significantly greater than Pre HBF levels (Table 1).

**TABLE 1:** Mean (± SEM) standing stature (*n* = 12) with 95% confidence intervals (CI) at Pre HBF, immediately after 4 h of supine hyper‐buoyancy flotation (Post HBF) and following 15 min of upright 1 *g* exposure (Post 1G).

Standing stature (cm)	Pre HBF	Post HBF	Post 1G	Repeated measures ANOVA
Mean	173.5 ± 2.5	175.1 ± 2.5	174.8 ± 2.5	*F* (2,22) = 110.283, *P *< 0.001^*^
95% CI	168.1–178.9	169.6–180.7	169.3–180.3	

* Statistically significant difference between conditions (*P* < 0.05).

### 3.2 Cervical IVD height

Cervical IVD image quality was satisfactory at all levels (C3–T1). Cervical (anterior) IVD height increased significantly across all levels following 4 h HBF (Pre HBF vs. HBF4). Subsequent 1 *g* exposure reduced lower cervical IVD height (C6–T1) by approximately 9%, although values remained above Pre HBF levels (Table 2).

**TABLE 2:** Mean (±SEM) cervical anterior intervertebral disc (IVD) height (*n* = 12) with 95% confidence intervals (CI) at Pre HBF, Post HBF (after 4 h of supine hyper‐buoyancy flotation) and Post 1G (after 15 min of upright 1 *g* exposure).

IVD level (cm)	Pre HBF	HBF4	Post 1G	Repeated measures ANOVA
C3–C4	0.44 ± 0.03	0.49 ± 0.03 (+11%)	0.48 ± 0.03 (−3%)	*F* (2,22) = 2.632, *P* = 0.094
95% CI	0.37–0.51	0.42‐0.54	0.42–0.54	
C4–C5	0.45 ± 0.03	0.52 ± 0.02 (+15%)	0.48 ± 0.02 (−8%)	*F* (2,22) = 8.965, *P* = 0.001^*^
95% CI	0.39–0.50	0.47–0.55	0.42–0.53	
C5–C6	0.45 ± 0.02	0.49 ± 0.02 (+8%)	0.48 ± 0.02 (−3%)	*F* (2,22) = 2.859, *P* = 0.079
95% CI	0.40–0.49	0.44–0.53	0.43–0.52	
C6–C7	0.43 ± 0.02	0.51 ± 0.02 (+24%)	0.45 ± 0.02 (−12%)	*F* (2,22) = 23.544, *P *< 0.001^*^
95% CI	0.39–0.46	0.46–0.55	0.42–0.48	
C7–T1	0.39 ± 0.02	0.47 ± 0.02 (+20%)	0.44 ± 0.02 (−7%)	*F* (2,22) = 14.265, *P *< 0.001^*^
95% CI	0.33–0.43	0.41–0.52	0.39–0.48	

* Data are presented for each cervical level (C3–T1). ^*^Significant difference between conditions (*P* < 0.05).

Cervical IVD height increased progressively during HBF across all levels (C3–T1). At 2 h (HBF2), increases were evident at C3–C4 and C7–T1 only, whereas at HBF4, IVD height was increased across all levels compared with HBF0 (Table 3).

**TABLE 3:** Mean (± SEM) cervical (C3–T1) anterior intervertebral disc (IVD) height (*n* = 12) with 95% confidence intervals (CI) measured during HBF at HBF0, HBF2 and HBF4.

IVD level (cm)	HBF0	HBF2	HBF4	Repeated measures ANOVA
C3–C4	0.43 ± 0.02	0.47 ± 0.02 (+9%)	0.49 ± 0.03 (+13%)	*F* (2,22) = 5.397, *P* = 0.012^*^
95% CI	0.38–0.46	0.42–0.50	0.42–0.54	
C4–C5	0.45 ± 0.02	0.46 ± 0.02 (+2%)	0.52 ± 0.02 (+15%)	*F* (2,22) = 11.662, *P *< 0.001^*^
95% CI	0.40–0.50	0.42–0.50	0.47–0.55	
C5–C6	0.44 ± 0.02	0.46 ± 0.02 (+4%)	0.49 ± 0.02 (+11%)	*F* (2,22) = 14.558, *P *< 0.001^*^
95% CI	0.38–0.48	0.40–0.50	0.44–0.53	
C6–C7	0.44 ± 0.02	0.47 ± 0.02 (+6%)	0.51 ± 0.02 (+15%)	*F* (2,22) = 11.066, *P *< 0.001^*^
95% CI	0.39–0.48	0.42–0.51	0.46–0.55	
C7–T1	0.39 ± 0.02	0.45 ± 0.02 (+15%)	0.47 ± 0.02 (+20%)	*F* (2,22) = 20.471, *P *< 0.001^*^
95% CI	0.34–0.43	0.40–0.49	0.41–0.52	

* Significant effect of HBF (*P* < 0.05).

### 3.3 Semispinalis cervicis and longus colli muscle CSA and muscle thickness

There was no effect of load on the CSA or thickness of semispinalis cervicis or longus colli (Table 4).

**TABLE 4:** Mean (± SEM) muscle cross‐sectional area (CSA) and thickness (*n* = 12) with 95% confidence intervals (CI) for longus colli and semispinalis cervicis at Pre HBF, Post HBF (after 4 h of supine hyper‐buoyancy flotation) and Post 1G (after 15 min of upright 1 *g* exposure).

Level	Pre HBF	HBF4	Post 1G	Repeated measures ANOVA
Longus colli CSA	0.96 ± 0.11	0.95 ± 0.08	0.96 ± 0.10	*F* (2,22) = 0.018, *P* = 0.982
95% CI	0.70–1.22	0.75–1.14	0.72–1.20	
Longus colli thickness	0.75 ± 0.05	0.77 ± 0.04	0.79 ± 0.04	*F* (2,22) = 2.659, *P* = 0.092
95% CI	0.64–0.87	0.68–0.87	0.70–0.89	
Semispinalis CSA	1.45 ± 0.19	1.31 ± 0.14	1.39 ± 0.14	*F* (2,22) = 0.399 *P* = 0.676
95% CI	1.03–1.87	1.00–1.62	1.07–1.71	
Semispinalis thickness	0.67 ± 0.04	0.68 ± 0.04	0.70 ± 0.04	*F* (2,22) = 0.210 *P* = 0.812
95% CI	0.56–0.78	0.60–0.77	0.60–0.79	

### 3.4 Vertebral stiffness

Across the entire column, passive vertebral stiffness decreased following 4 h HBF (HBF0 vs. HBF4). Specifically, mid‐ and lower cervical and lumbar regions were decreased, with no changes in the upper cervical or thoracic regions (Table 5). Subsequent 1 *g* exposure (HBF4 vs. Post 1G) increased stiffness in the lower cervical region only, with no changes in the remaining regions. Despite this partial recovery, stiffness remained lower than Pre HBF levels (Pre HBF vs. Post 1G) across the entire column and in the mid‐ and lower cervical regions.

**TABLE 5:** Mean (± SEM) passive vertebral stiffness (*n* = 12) with 95% confidence intervals (CI) across the entire spinal column and by region (upper cervical, mid‐cervical, lower cervical, thoracic, lumbar) at Pre HBF, HBF0, HBF4 and Post 1G (after 15 min of upright 1 *g* exposure).

Level (N)	Pre HBF	HBF0	HBF4	Post 1G	Repeated measures ANOVA
Entire column	76.41 ± 0.44	76.35 ± 0.62	71.84 ± 0.62	72.52 ± 0.50	*F* (3,399) = 33.821, *P *< 0.001^*^
95% CI	75.54–77.28	75.12–77.58	70.62–73.07	71.52–73.51	
Upper cervical	75.00 ± 1.72	73.99 ± 1.67	67.27 ± 1.08	70.83 ± 2.20	*F* (3,33) = 5.843, *P* = 0.003^*^
95% CI	71.21–78.80	70.31–77.68	64.88–69.67	65.97–75.69	
Mid cervical	78.00 ± 1.12	73.85 ± 2.26	67.77 ± 1.85	72.55 ± 1.61	*F* (3,33) = 12.762, *P *< 0.001^*^
95% CI	75.52–80.49	68.86–78.84	63.70–71.84	69.00–76.09	
Lower cervical	79.55 ± 1.33	75.09 ± 1.92	67.48 ± 1.40	74.71 ± 0.92	*F* (3,33) = 16.943, *P *< 0.001^*^
95% CI	76.61–82.50	70.85–79.33	64.38–70.57	72.67–76.75	
Thoracic	76.00 ± 1.14	76.50 ± 1.60	70.08 ± 1.87	70.92 ± 1.59	*F* (3,33) = 5.257, *P* = 0.004^*^
95% CI	73.49–78.51	72.96–80.04	65.95–74.21	67.42–74.41	
Lumbar	76.67 ± 1.16	80.08 ± 1.41	70.58 ± 2.51	72.4 ± 1.88	*F* (3,33) = 6.412, *P* = 0.002^*^
95% CI	74.12–79.21	76.97–83.20	65.05–76.11	68.29–76.55	

^*^Significant difference between conditions (*P* < 0.05).

### 3.5 Neck pain ratings

No participant reported severe (≥6) neck pain. Most participants (10/12) reported mild discomfort and/or spinal stiffness during HBF, which entirely resolved after 15 min of 1 *g* exposure (Table 6). Neck pain increased at HBF2 and HBF4 vs. HBF0, with no difference between HBF2 and HBF4. Pain was reduced after 1 *g* exposure (HBF4 vs. Post 1G), and no longer differed from Pre HBF levels.

**TABLE 6:** Mean (± SEM) neck pain ratings (*n* = 12) with 95% confidence intervals (CI) at Pre HBF, HBF0, HBF2, HBF4 and Post 1G (after 15 min of upright 1 *g* exposure).

Neck pain (0/10)	Pre HBF	HBF0	HBF2	HBF4	Post 1G	Repeated measures ANOVA
Mean	0 ± 0.00	0 ± 0.00	1.42 ± 0.35	1.95 ± 0.31	0.12 ± 0.09	*F* (2,22) = 110.283, *P *< 0.001^*^
95% CI	0–0	0–0	0–4.50	0–4.5	0–1	

^*^Significant difference between conditions (*P* < 0.05).

### 3.6 Correlations

No significant correlations were observed between neck pain ratings and changes in standing stature (*R* = −0.328, *P* = 0.223) or cervical IVD height changes at any level (C3–T1; all *P* > 0.05). Neck pain at HBF4 was positively correlated with mid‐cervical passive vertebral stiffness (*R* = 0.587, *P* = 0.045), but not with stiffness across the entire column, or within any other regions. No significant correlations were observed between neck pain reduction at Post 1G and any other Post 1G parameter.

## 4 DISCUSSION

HBF increased standing stature by 1.6 cm (+1.0%), which was significantly attenuated following 15 min of 1 *g* exposure, but remained above Pre HBF levels. Cervical anterior IVD height at all levels (C3–T1) was significantly increased at HBF4 (vs. Pre HBF). Subsequent 1 *g* exposure induced a significant reduction in the lower cervical column height (C6–C7, C7–T1) that remained greater than Pre HBF. No changes in the thickness or CSA of longus colli or semispinalis cervicis were observed. In contrast, passive vertebral stiffness was significantly decreased at HBF4 across the entire column, mid‐cervical, lower cervical and lumbar, but not upper cervical or thoracic regions. 1 *g* exposure increased stiffness only in the lower cervical region, but not across the entire column, upper cervical, mid‐cervical, thoracic or lumbar regions. Mild (1.5/10) neck pain was induced at 2 h HBF, which was maintained at HBF4. Neck pain was significantly reduced after 15 min exposure but not entirely ameliorated. No significant correlations between neck pain rating and changes in standing stature, nor any cervical IVD height changes were observed. A positive correlation between mid‐cervical passive vertebral stiffness and induced neck pain was reported, but this was not observed following 1 *g* exposure.

### 4.1 Stature

All participants completed the 4 h HBF and subsequent 15‐min 1 *g* sessions without issue. Similar stature increases were induced by 4‐h HBF as those reported in previous 4 h HBF studies (Carvil, Russomano, Jones et al., 2017; Marcos‐Lorenzo et al., 2024) albeit lower than induced by 8 h HBF (2.1 ± 0.2 cm) (Breen et al., 2023). However, each study appears to possess broadly similar levels of intra‐individual variability. In fact, whilst stature increments (even with 8 h HBF) are lower than some of those reported inflight (Brown, 1977), the validity of historic inflight measures has been questioned (Green & Scott, 2018). Thus, HBF appears to be a robust and reliable microgravity ground‐based analogue of microgravity‐induced stature increments (and back pain).

Recent inflight data suggest that astronaut stature increases until around flight‐day 15, before plateauing (Young et al., 2021). On this basis, investigation of HBF‐induced stature increments during exposures longer than 8 h to be compared with analogous assessments in space are warranted (Green & Scott, 2018).

Following 8 h HBF, provision of 15 min of 1 *g* exposure significantly albeit modestly (∼−20%) ameliorated induced stature increments. Such ‘re‐compression’ is similar to that induced by ∼0.2 Gz axial loading provided by donning a Mk VI SkinSuit (Breen et al., 2023). It is also similar to a recent study demonstrating that 15 min of 1 *g*, with or without 30 s of 50% bodyweight static loading, was able to ameliorate ∼40% HBF‐induced stature increments (Marcos‐Lorenzo et al., 2024). Anecdotally, on the ISS rapid stature reductions to enable EVA suit fit have been generated by the unprescribed use of the ARED to provide large axial impulse loads – although it has been suggested this may contribute to post‐flight spinal (including IVD) pathology (Green & Scott, 2018). Thus, further studies are warranted to determine axial reloading dynamics to inform definition of optimal magnitudes and duration along with potential risks.

### 4.2 Cervical intervertebral disc height

In our study, good quality ultrasonic images of the entire cervical IVD spine (C3–T1) were acquired. At 2 h HBF only C7–T1 and C3–C4, that is, lower cervical IVDs, were significantly expanded – consistent with IVD expansion being potentially a key driver of early mission stature increases (Sayson & Hargens, 2008), and back pain (Pool‐Goudzwaard et al., 2015). Four hours of HBF induced significant and progressive increases in cervical IVD height across all levels (C3–T1), ranging from 11% at C5–C6 to 20% at C7–T1. These findings are consistent with a progressively greater effect of gravitational loading upon IVD geometry as one moves caudally down the spine (Putzer et al., 2016). Indeed, cervical IVD swelling magnitude was less than that we reported for the lumbar region (Marcos‐Lorenzo et al., 2024). Whilst this contrasts with a failure to observe IVD height changes with ultrasound on the ISS (Garcia et al., 2018), this may reflect not only variability in stature increments, but also the fact ultrasound is extremely challenging in microgravity. Thus, further evaluation of cervical IVD height (in conjunction with stature assessment) in microgravity is warranted.

In our study, consistent with stature changes, increments in IVD height induced by 4 h HBF were significantly attenuated – albeit not entirely ameliorated – by 15 min of 1 *g* exposure. Whilst lower cervical (C6–T1) IVD height remained significantly greater (9%) than Pre HBF, upper cervical IVD height (C3–C6) no longer differed (3%) from pre, indicative of maximal reduction of C6–C7 (−12%) and minimal at C3–C4 (−3%), respectively. Increases in stature diminished by 22% and cervical IVD expansion was similarly attenuated – indicating an incomplete yet substantial reversal of HBF‐related changes. As IVD (height) compression was modest, it appears that longer exposure to 1 *g* is required. The temporal profile of cervical IVD height recovery after re‐exposure to 1 *g* therefore warrants further investigation. Although ultrasound is less sensitive than MRI, it has been shown to detect dynamic IVD height changes under relevant loading conditions, including supine trunk exercise with 1 *g* loading applied at the centre of mass via SAHC (Marcos‐Lorenzo et al., 2022).. Interestingly, our recent study showed no supplementary effect of 50% BW loaded trunk flexion on IVD height compared to 15 min of 1 *g* exposure (Marcos‐Lorenzo et al., 2024). However, dynamic lumbar IVD compression is reported in response to gravitational loading associated with re‐orientation (Belavý et al. 2011), diurnal activity patterns (Ledsome et al. 1996) and exercise (Kingsley et al. 2012) in 1 *g*. Whilst not acting directly on the cervical spine, loading at the shoulder through 30 s of 50% BW vertebral dynamic loading partially ameliorated cervical IVD height expansion, albeit to a greater extent at L5–S1 than L2–L3 (Marcos‐Lorenzo et al., 2024), suggestive that loading sharing may have complex effects along the spine (Breen et al., 2023).

Re‐introduction of 1 *g* appears to be a mild compressive cervical IVD stimulus, suggesting that of the use of ARED or T2 is likely to affect the cervical spine. Furthermore, in microgravity repeated neck hyperextension when orientating within nodes and translating may also affect cervical IVD mechanics (He et al., 2025). This complex picture must also consider that the provision of periodic axial loading upon IVDs is also key to IVD regulation (Malko et al., 2002) via membrane water and protein (e.g., collagens and proteoglycans) transport (McMillan et al., 1996). Thus, determination of adequate and appropriate inflight axial loading may be key to reducing cervical vulnerability during re‐entry and landing (Green & Scott, 2018).

Moderate ‘progressive’ axial loading (Waldie et al. 2011) via the donning of tight‐fitting textiles that promote spinal control on Earth (Rathinam et al., 2013) such as the 0.2 Gz equivalent Mk VI SkinSuit has been proposed on the basis of ameliorating and reversing stature increments (Green & Scott, 2018) induced by HBF unloading. In fact, the Mk VI SkinSuit has been implemented on the ISS (Stabler et al., 2017). However, its effect upon the spine in orbit has not been published. As such the effect of axial loading and determination of its effects and optimal ‘dose’ (i.e., interaction of magnitude and time) warrant further investigation.

### 4.3 Effects on semispinalis and longus colli thickness and CSA

Neither 4 h HBF nor 15 min of 1 *g* exposure had any observable (by ultrasound) effect upon semispinalis cervicis and longus colli thickness or CSA, which is perhaps unsurprising given the short duration of unloading. This absence also means that there were no detectable changes in CSA or muscle thickness after 1 *g* exposure. An absence of significant changes with the re‐introduction of 1 *g* suggests, at least in terms of cervical CSA and muscle thickness, that re‐entry, landing and re‐ambulation may have only a modest effect, supporting the validity of post‐flight vertebral MRI as an index of inflight changes (Bailey et al., 2022). The spatial resolution of ultrasound (compared to MRI) may be an issue. However, the absence of morphological change does not imply preserved muscle function, as neuromuscular adaptations may occur that are not detectable with ultrasound. For instance, changes in muscle activation patterns or stiffness can precede structural alterations (Fitts et al., 2007; Narici & de Boer et al., 2011). Thus, future studies should incorporate assessment of muscle activation (fine wire or high‐density EMG) and assessment of muscle (or tissue) stiffness.

Interestingly, long‐duration spaceflight is not associated with neck muscle atrophy (LeBlanc et al., 2000), which may relate to the loading associated with exercise, particularly ARED. If that is the case, vertebral stiffness may be a more sensitive measure.

### 4.4 Vertebral stiffness

Passive vertebral stiffness was significantly decreased across the entire column, mid‐cervical, lower cervical and lumbar regions at HBF4 compared to HBF0, thereby accounting for modulation resulting from the transition from standing to adopting a supine posture. Such reductions suggest that HBF allows adoption of a neutral neck position with minimal requirement for head stabilisation, although assessment of paraspinal EMG is required to confirm this (Swanenburg et al., 2020). In contrast, no changes in upper cervical or thoracic region stiffness were observed. Whilst the thoracic vertebrae may have more consistent stiffness by virtue of being fixed to the ribs and sternum (Hofstetter et al., 2018), the rationale for the failure to observe upper cervical changes is unknown, although unloading‐induced modulation of load sharing pattern may once again play a key role (Breen et al., 2023). Changes in load sharing and spinal kinematics (Bailey et al., 2022) would be consistent with that associated with spaceflight, albeit assumed to relate to spinal flattening (Andreoni et al., 2000).

Indeed, recent data suggest that even transient microgravity induced by parabolic flight can induce significant upper lumbar and lower thoracic spine flattening (Meinke et al., 2025), together with modulation of multifidus and erector spinae activity that partly correlated with flattening. However, it must be noted that hypergravity (1.8 *g*) preceding and following microgravity may have a role. Unfortunately, we were unable to assess spinal curvature in this study. Therefore, any interpretation regarding spinal curvature flattening should be considered speculative and requires confirmation using direct measures of spinal alignment, for example standardized standing lateral whole‐spine radiographs (Vialle et al., 2005) or biplanar EOS imaging (Kim et al., 2018); MRI‐based assessment may also be useful, although differences between supine MRI and standing radiographs should be considered (Hasegawa et al., 2018).

Fifteen minutes of 1 *g* following 4 h HBF significantly increased lower cervical stiffness close to pre‐levels. In contrast, significant stiffness recovery was not observed across the entire column, nor in the upper cervical, mid‐cervical, thoracic or lumbar regions, which is broadly in line with that reported by Hofstetter and co‐workers. However, Hofstetter and co‐workers also suggested that high axial forces can induce reductions in cervical vertebral stiffness due to capsular ligament laxity (buckling) effects (Hofstetter et al., 2021). In fact, passive vertebral stiffness remained lower than Pre HBF following 1 *g* across the entire column, mid‐cervical and lower cervical. However, this was not the case for upper cervical, thoracic or lumbar regions. Thus, investigations of higher and longer duration axial loading exposure post HBF‐induced unloading are warranted with larger sample sizes as significant (∼10–15%) inter‐individual variability in PulStar‐assessed vertebral stiffness has been reported (Häusler et al., 2020). Longer exposure to HBF and subsequent 1 *g* is also likely to have a more profound effect upon active (weight‐bearing) vertebral stiffness (Stokes & Gardner‐Morse, 2003) through modulation of both muscular (Hodges et al., 2013) and connective tissue (Stokes et al. 2003) contributions to vertebral stiffness.

Whether changes in vertebral stiffness translate into modulation of IVD pathology and/or prolapse is unclear but warrants consideration inflight given that cervical IVD pathology appears frequent (Garcia et al., 2018) and risk of IVD prolapse post‐flight is significant (Johnston et al., 2010). Whilst worrying on Earth, translation of cervical risk to that when landing on the lunar surface could be catastrophic as the weight of the EVA suit helmet will be significant (Lalwala et al., 2023).

### 4.5 Neck pain

None of the participants reported severe (≥6/10) neck pain during 4 h HBF, although several (10 out of 12) reported mild discomfort and/or spinal stiffness. The magnitude of cervical pain in comparable unloading analogues remains poorly characterised, as HDTBR studies have generally focused on back pain or overall pain rather than conducting a systematic assessment of cervical pain (Belavý et al., 2010; Hutchinson et al., 1995). However, the cervical region appears to be less affected (1.95 ± 0.3) than that previously reported in the lumbar region (2.90 ± 1.26) following 4 h HBF (Marcos‐Lorenzo et al., 2024). Furthermore, it appears less severe, albeit more prevalent (Wing et al., 1991) than that reported by crew inflight, (Pool‐Goudzwaard et al., 2015).

Interestingly, in our study neck pain did not differ between 2 and 4 h HBF. Whether neck pain is induced and plateaus earlier than in other regions warrants further investigation. However, our findings may simply reflect the modest sample size and variability in pain ratings. Furthermore, 4 h HBF‐induced neck pain was largely absent (12 out of 12) following 15 min of 1 *g* exposure. Thus, the temporal dynamics of neck pain and its amelioration warrants further study with more prolonged exposure to HBF, with larger sample sizes.

### 4.6 Correlation with neck pain

We explored candidate mechanisms that may underpin neck pain by examining correlations between HBF‐induced changes and neck pain. Surprisingly, no significant correlation was observed between neck pain and change in stature, or any (ultrasonic) cervical IVD anterior height measure. This finding casts doubt on the hypothesis that IVD swelling underpins back pain inflight (Sayson et al., 2013; Belavy et al., 2016). However, the low magnitude of cervical IVD swelling observed in our study and the relatively poor spatial sensitivity of ultrasound may have limited the ability to observe a significant association. Similarly, no association with muscle thickness or CSA was observed, although given that no significant changes in these parameters were reported following 4 h HBF, this is unsurprising. Whether this is the case with longer exposure to HBF is unknown as modulation of semispinalis cervicis and longus colli has been associated with chronic idiopathic neck pain on Earth (Looveren et al., 2021).

A significant positive correlation was reported between neck pain and mid‐cervical vertebral stiffness. However, no correlation with stiffness across the entire column, nor any other region, was observed. Unfortunately, participants were not asked to identify and rate neck pain within each individual cervical region. Thus, whether neck pain was predominantly experienced in or around the mid‐cervical region remains unknown. The mechanisms underlying changes in vertebral stiffness are unclear as we failed to identify any other associations, raising the possibility that cervical lordosis flattening may contribute to stiffness, and thus vertebral instability (Panjabi, 1992) and ultimately impaired spinal kinematics (Crevecoeur et al., 2010). However, it must be noted that these relationships remain speculative as spinal curvature (and active spinal kinematics) was not assessed.

Despite neck pain being almost entirely ameliorated, no significant correlation between reduction in neck pain and any other parameter following 15 min of 1 *g* was observed. Nevertheless, the relationship between vertebral stiffness and cervical neck pain requires further investigation.

### 4.7 Conclusion

Four hours of HBF increased stature and cervical IVD height, both of which were partially reduced after 15 min of upright sitting in 1 *g*. No changes in longus colli or semispinalis cervicis muscle thickness or CSA were observed. Passive cervical vertebral stiffness decreased following HBF and remained below Pre HBF levels after 1 *g* exposure. Mild neck pain induced by HBF was alleviated by 1 *g* exposure. A positive correlation between neck pain at HBF4 and passive mid‐cervical vertebral stiffness suggests that changes in vertebral column stabilisation may contribute to neck pain. The lack of association between neck pain and IVD expansion raises the possibility that vertebral flattening contributes, although this cannot be confirmed without direct curvature assessment. Therefore, future studies should include direct assessment of spinal curvature and investigate longer HBF exposures to better understand spinal adaptations to unloading.

## AUTHOR CONTRIBUTIONS

D. Marcos‐Lorenzo, D. A. Green, J. Swanenburg, A. Gil Martínez conceived and designed the study; D. Marcos‐Lorenzo, P. Hernández Bernal, and R. Fernández Corujo performed the data collection; D. Marcos‐Lorenzo, D. A. Green and J. Swanenburg analysed the data. D. Marcos‐Lorenzo and D. A. Green wrote the manuscript. All authors have read and approved the final version of this manuscript and agree to be accountable for all aspects of the work in ensuring that questions related to the accuracy or integrity of any part of the work are appropriately investigated and resolved. All persons designated as authors qualify for authorship, and all those who qualify for authorship are listed.

## CONFLICT OF INTEREST

The authors declare no conflicts of interest.

## Data Availability

The data sets generated during and/or analysed during the current study are available at Figshare (https://doi.org/10.6084/m9.figshare.30896432) and from the corresponding author on reasonable request.
